# Supplementary figures and images for: Senescent epithelial cells remodel the microenvironment for the progression of oral submucous fibrosis through secreting TGF-β1
Source: PeerJ. 2023 Apr 19;11:e15158. doi: 10.7717/peerj.15158 (PMC10122456; doi:10.7717/peerj.15158)

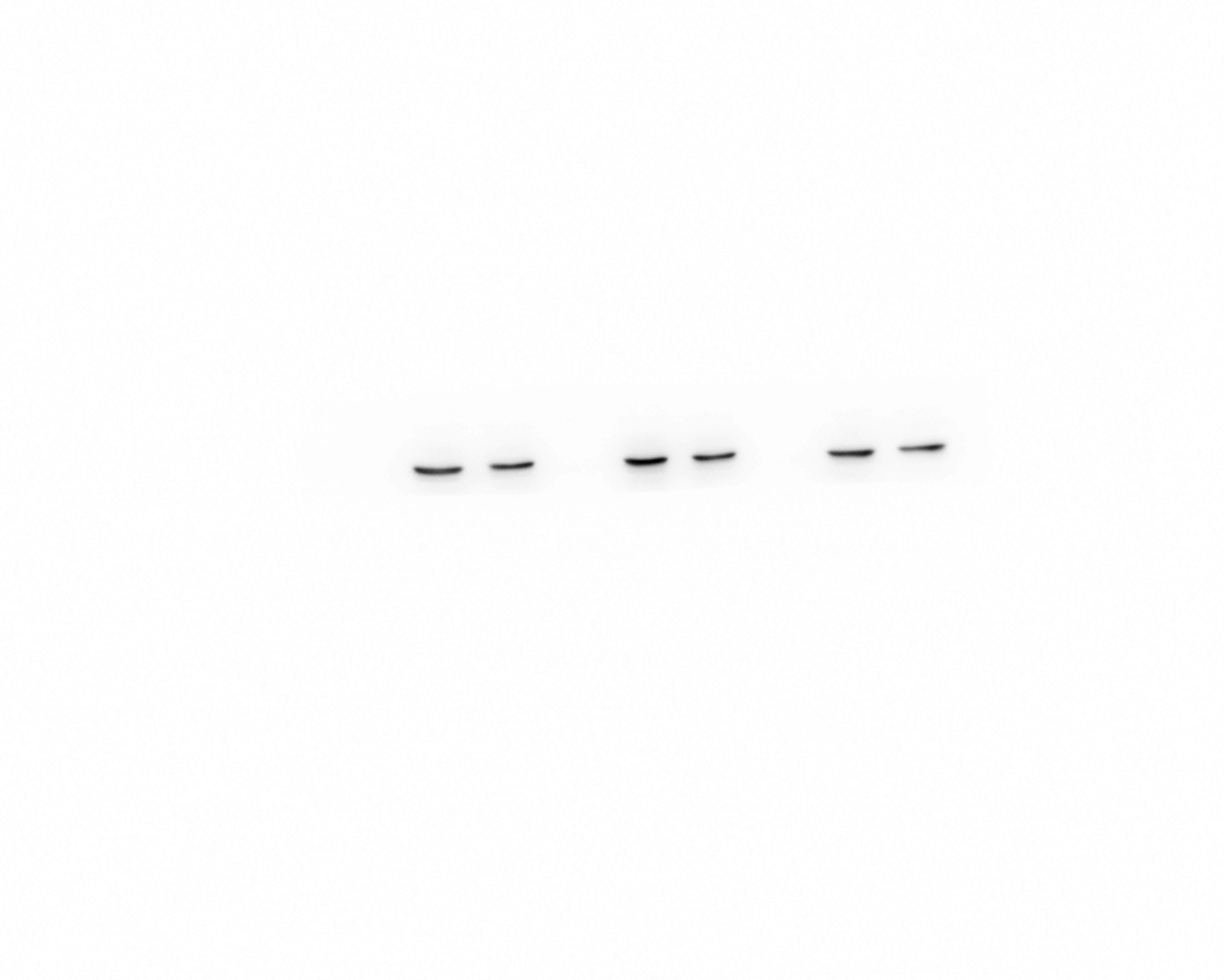

Supplement: Supplemental Information 6 — Total protein (35 µg) was extracted from the negative control HOKs (lane 1, 3, 5) and senescent HOKs (lane 2, 4, 6) (lanes were ordered from left to right). GAPDH (35kDa). [file peerj-11-15158-s006.tif]

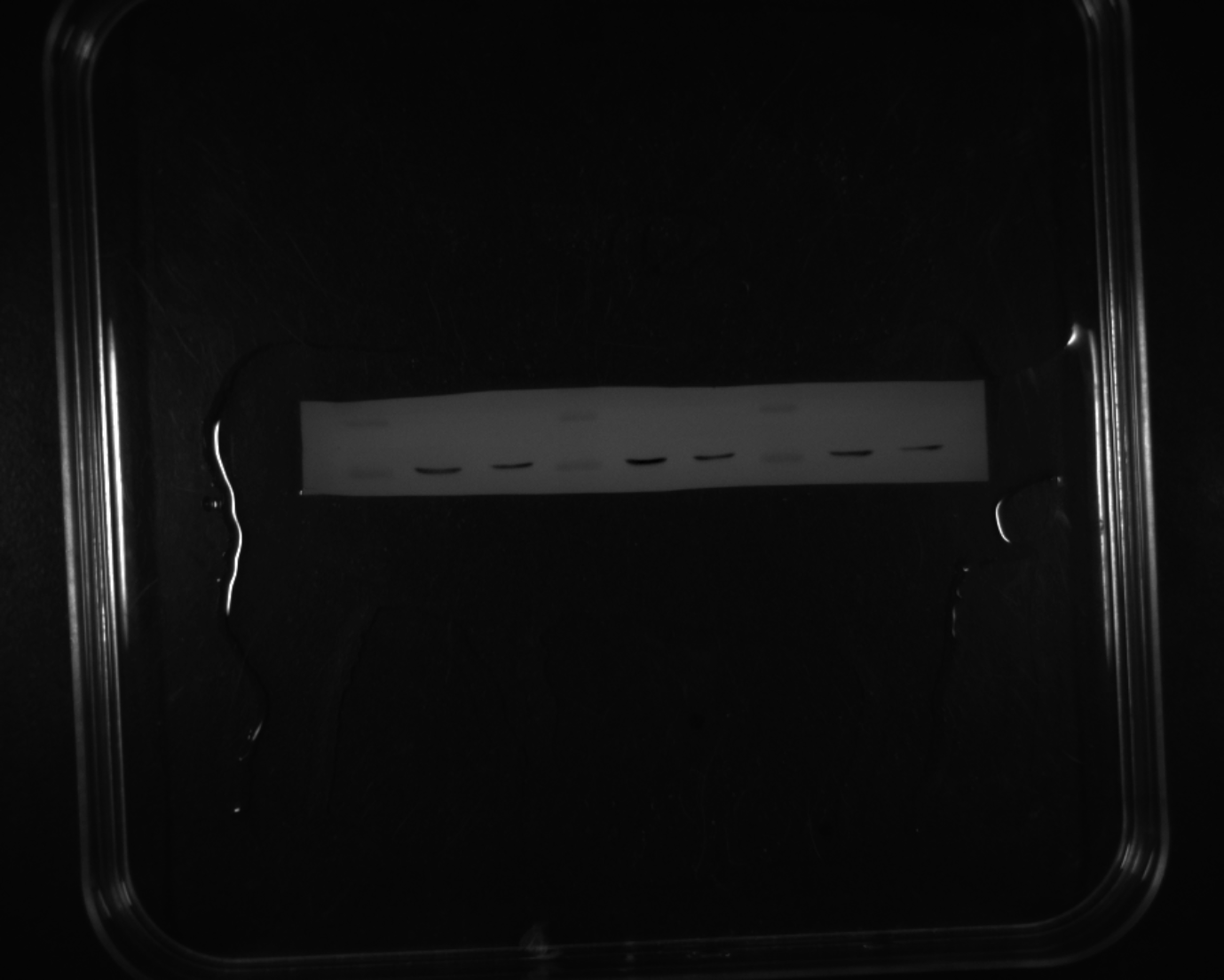

Supplement: Supplemental Information 7 — Total protein (35 µg) was extracted from the negative control HOKs (lane 1, 3, 5) and senescent HOKs (lane 2, 4, 6) (lanes were ordered from left to right). GAPDH (35 kDa). [file peerj-11-15158-s007.tif]

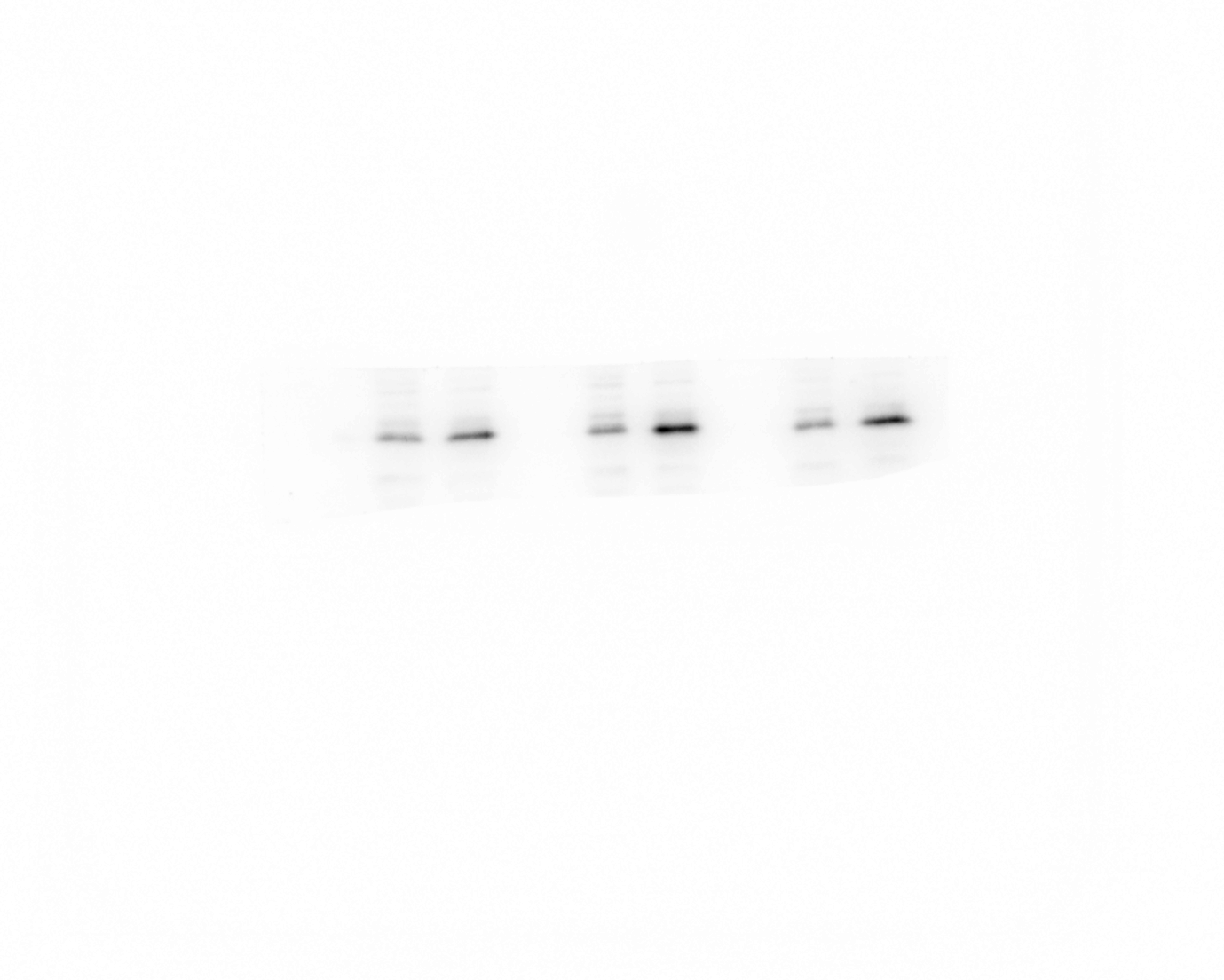

Supplement: Supplemental Information 8 — Total protein (35 µg) was extracted from the negative control HOKs (lane 1, 3, 5) and senescent HOKs (lane 2, 4, 6) (lanes were ordered from left to right). p21 (21 kDa). [file peerj-11-15158-s008.tif]

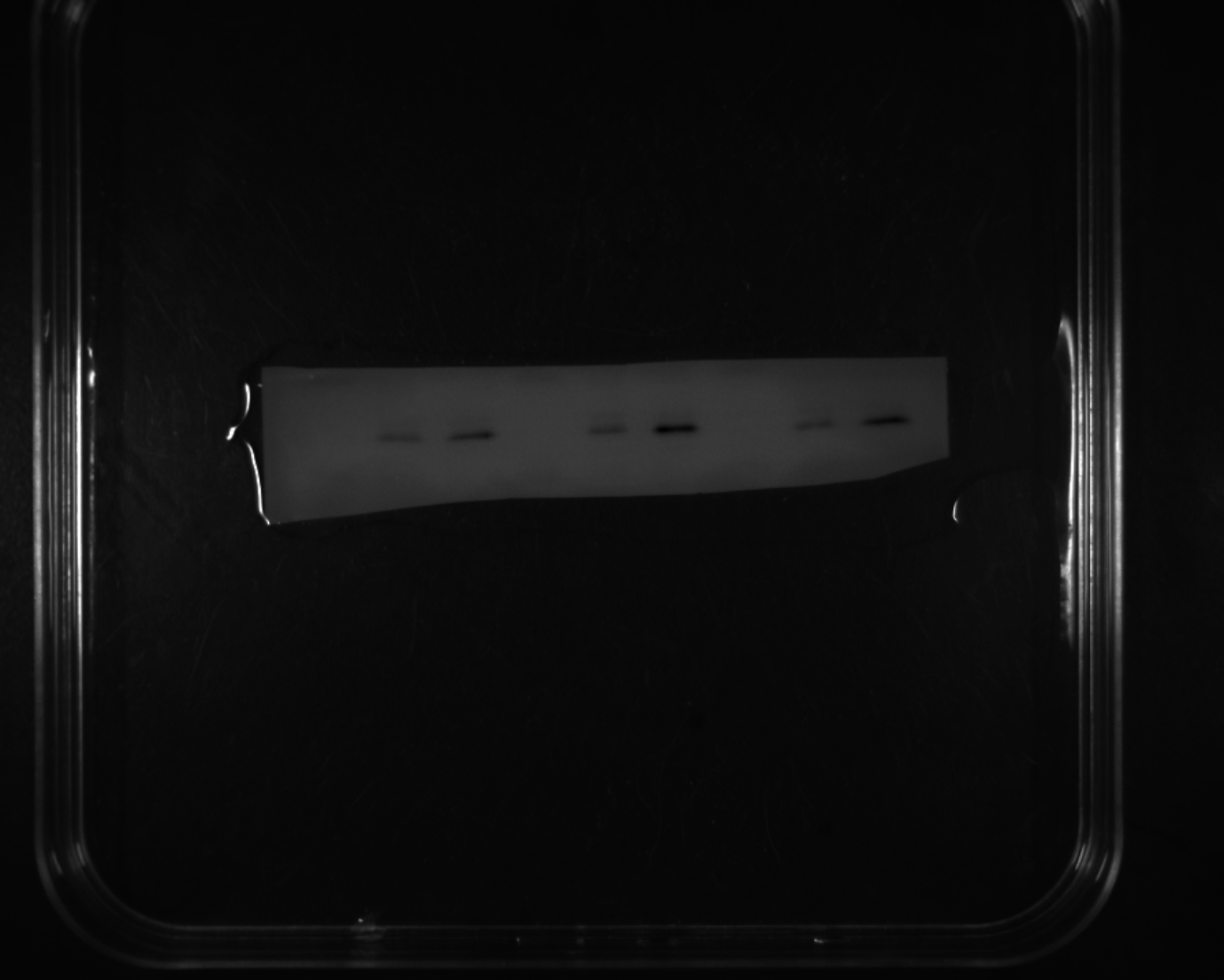

Supplement: Supplemental Information 9 — Total protein (35 µg) was extracted from the negative control HOKs (lane 1, 3, 5) and senescent HOKs (lane 2, 4, 6) (lanes were ordered from left to right). p21 (21 kDa). [file peerj-11-15158-s009.tif]

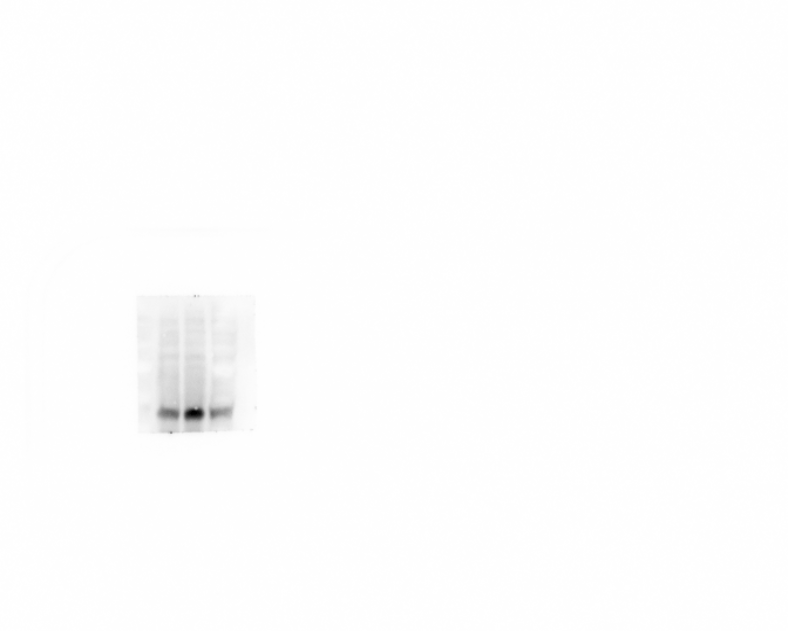

Supplement: Supplemental Information 10 — Total protein (35 µg) was extracted from the negative control HOKs (lane 1, 3) and senescent HOKs (lane 2) (lanes were ordered from left to right). TGF- β1 (44 kDa). [file peerj-11-15158-s010.tif]

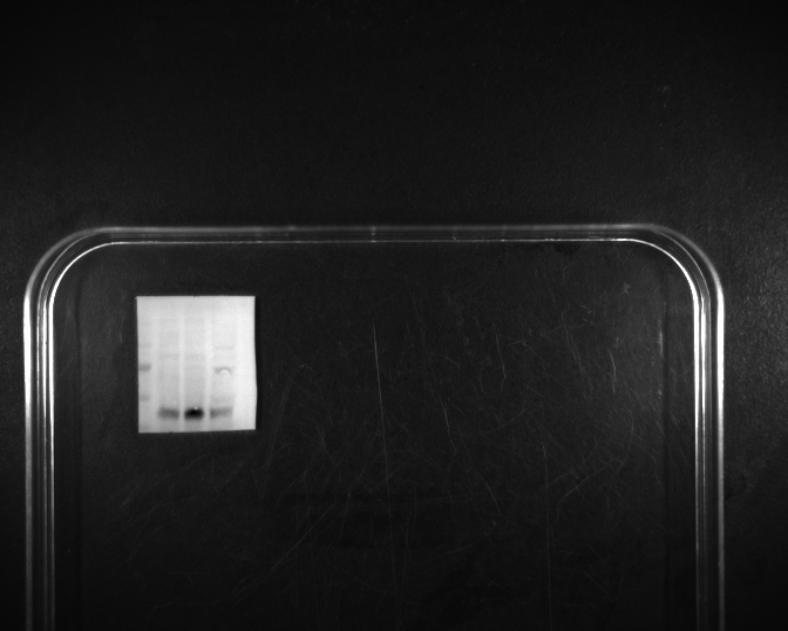

Supplement: Supplemental Information 11 — Total protein (35 µg) was extracted from the negative control HOKs (lane 1, 3) and senescent HOKs (lane 2) (lanes were ordered from left to right). TGF- β1 (44 kDa). [file peerj-11-15158-s011.tif]

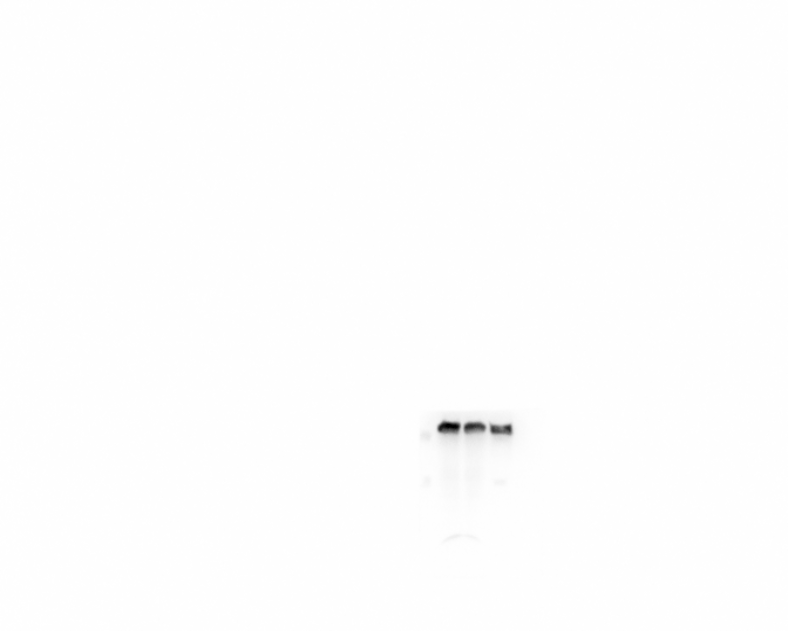

Supplement: Supplemental Information 12 — Total protein (35 µg) was extracted from the negative control HOKs (lane 1, 3) and senescent HOKs (lane 2) (lanes were ordered from left to right). GAPDH (35 kDa). [file peerj-11-15158-s012.tif]

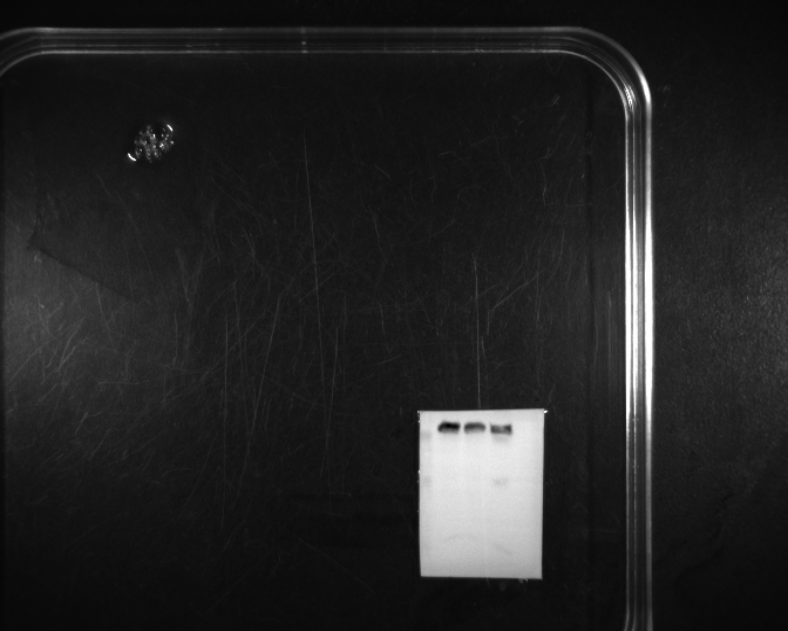

Supplement: Supplemental Information 13 — Total protein (35 µg) was extracted from the negative control HOKs (lane 1, 3) and senescent HOKs (lane 2) (lanes were ordered from left to right). GAPDH (35 kDa). [file peerj-11-15158-s013.tif]

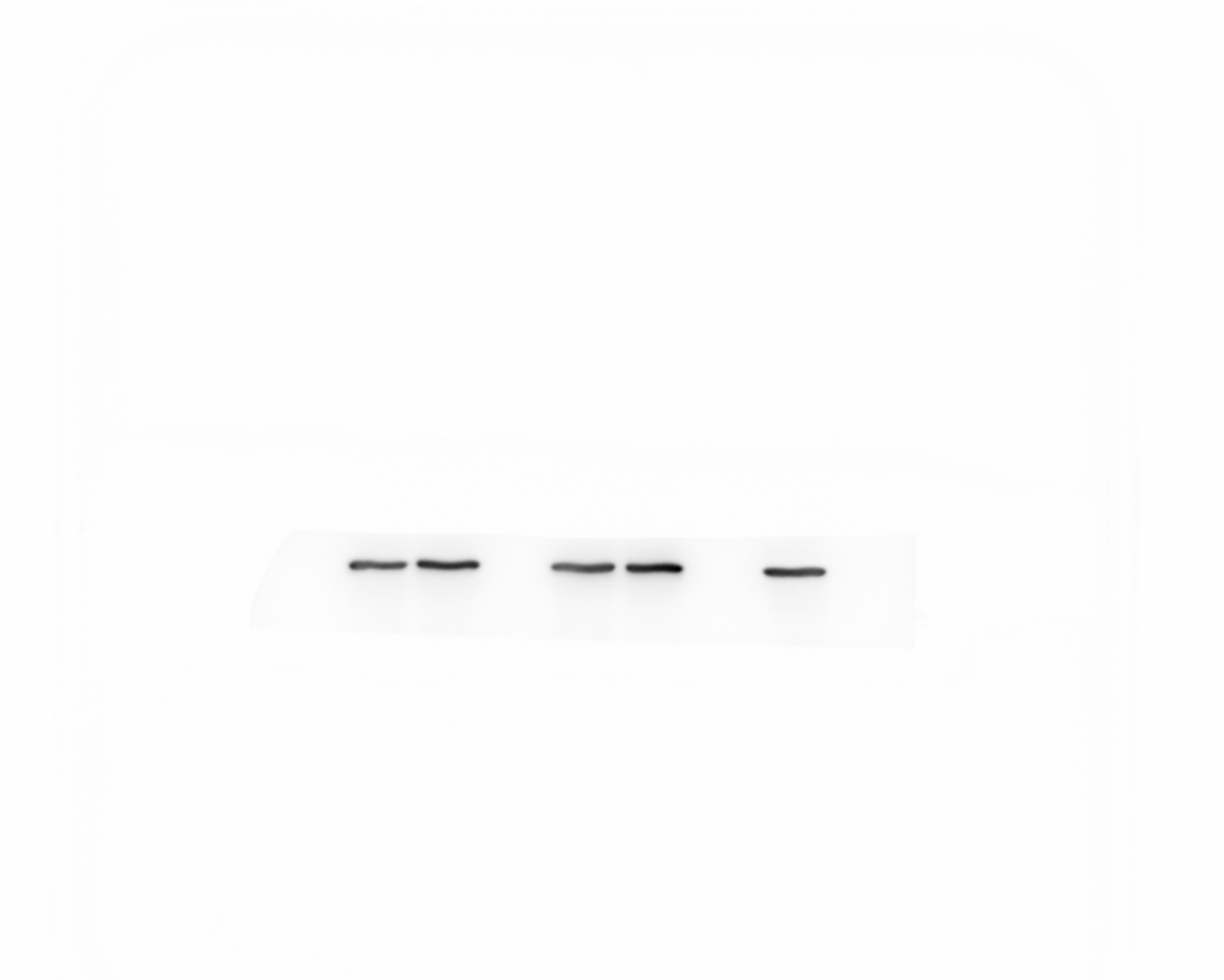

Supplement: Supplemental Information 14 — Total protein (35 µg) was extracted from the negative control HOKs (lane 1, 3, 4) and senescent HOKs (lane 2, 5) (lanes were ordered from left to right). GAPDH (35 kDa). [file peerj-11-15158-s014.tif]

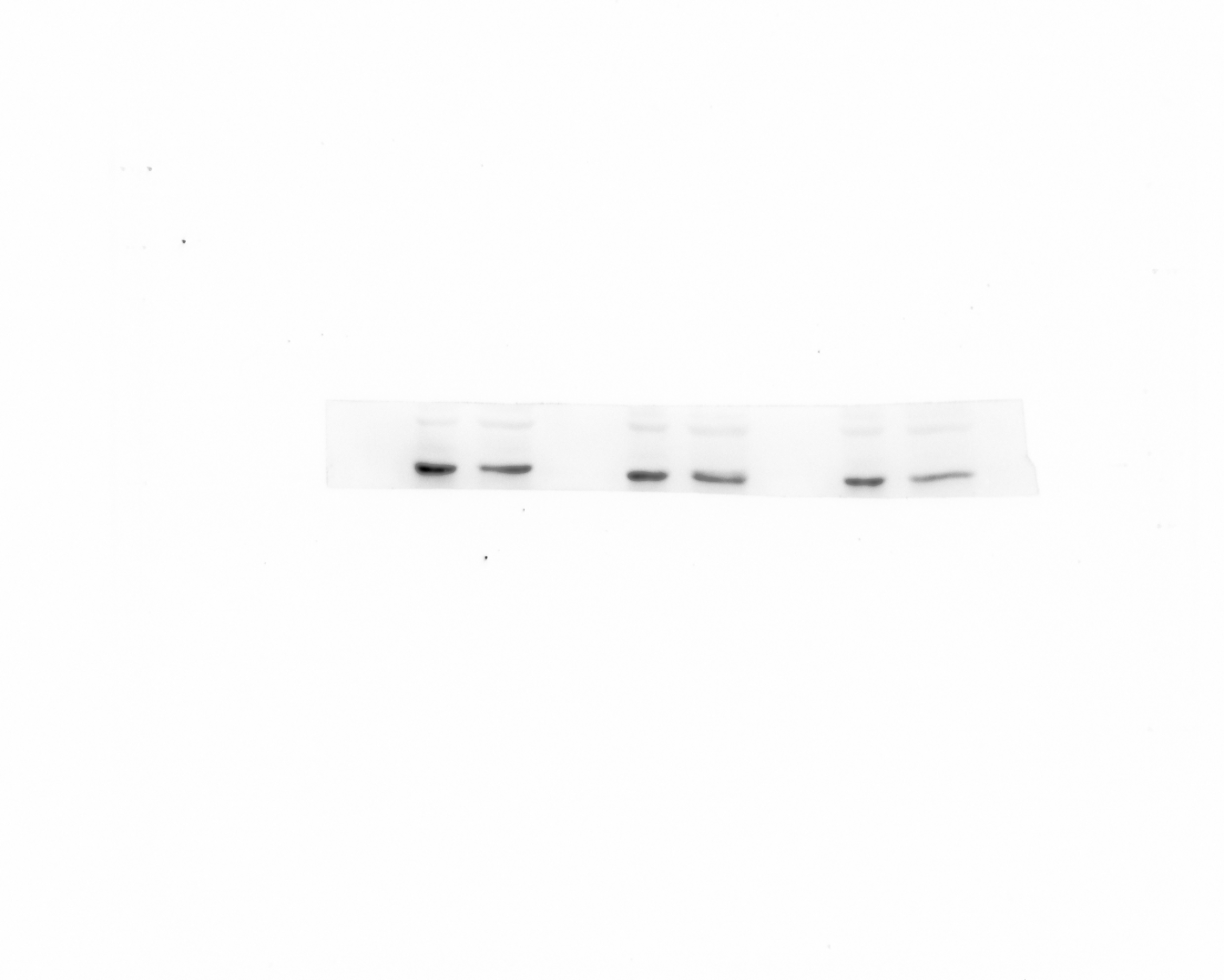

Supplement: Supplemental Information 15 — Total protein (35 µg) was extracted from the negative control HOKs (lane 1, 3, 5) and senescent HOKs (lane 2, 4, 6) (lanes were ordered from left to right). GAPDH (35 kDa). [file peerj-11-15158-s015.tif]

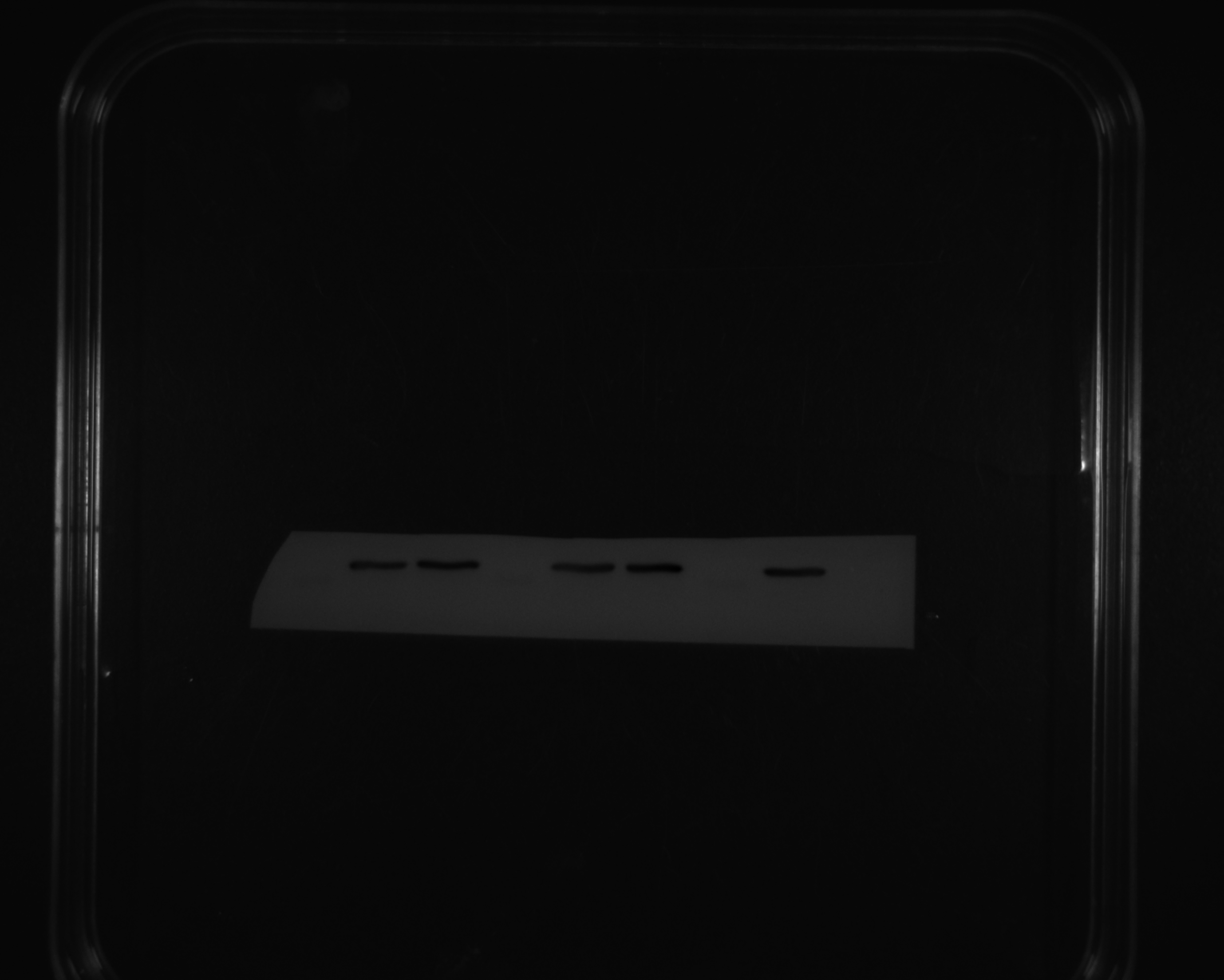

Supplement: Supplemental Information 16 — Total protein (35 µg) was extracted from the negative control HOKs (lane 1, 3, 4) and senescent HOKs (lane 2, 5) (lanes were ordered from left to right). GAPDH (35 kDa). [file peerj-11-15158-s016.tif]

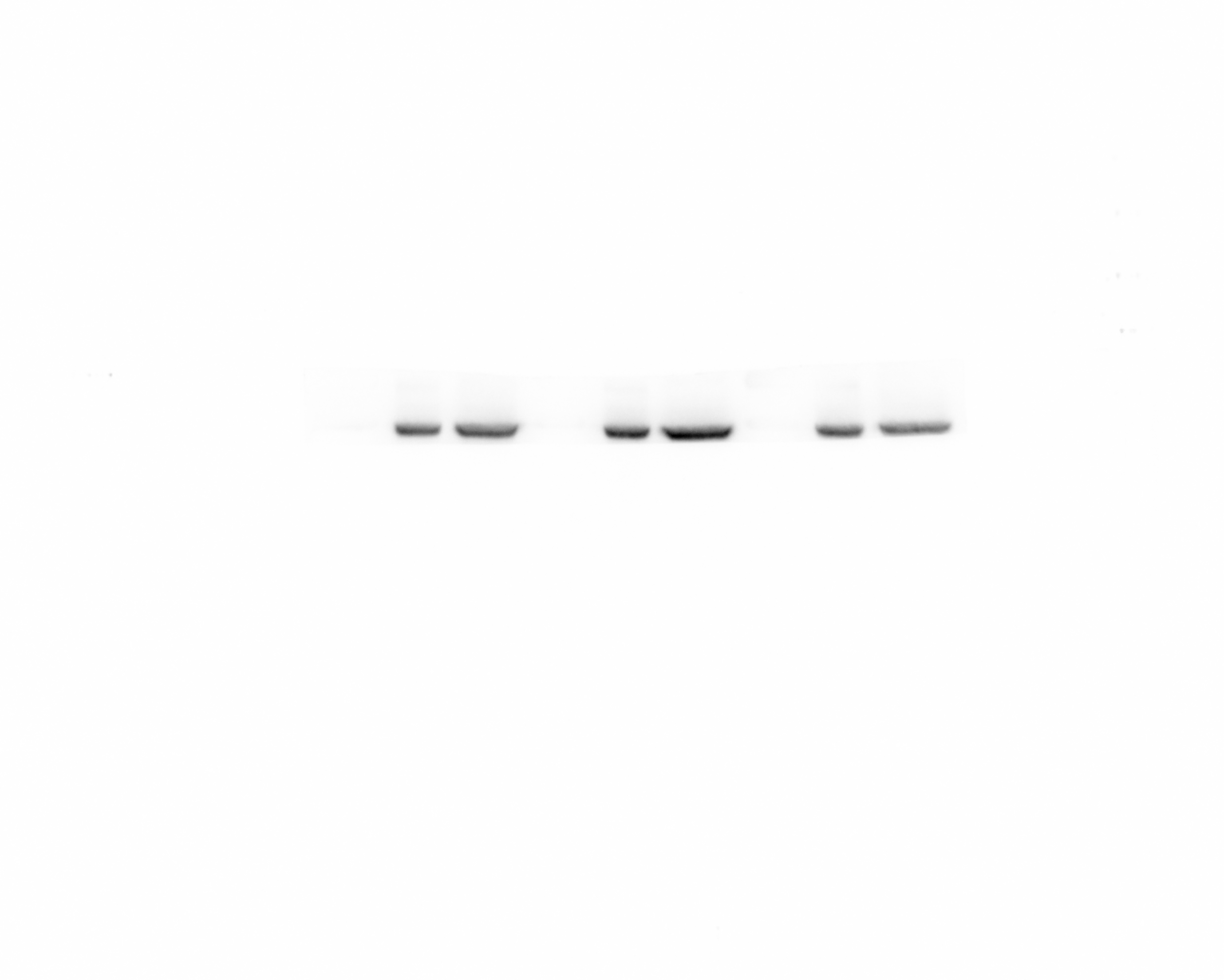

Supplement: Supplemental Information 17 — Total protein (35 µg) was extracted from the negative control HOKs (lane 1, 3, 5) and senescent HOKs (lane 2, 4, 6) (lanes were ordered from left to right). p53 (53 kDa). [file peerj-11-15158-s017.tif]

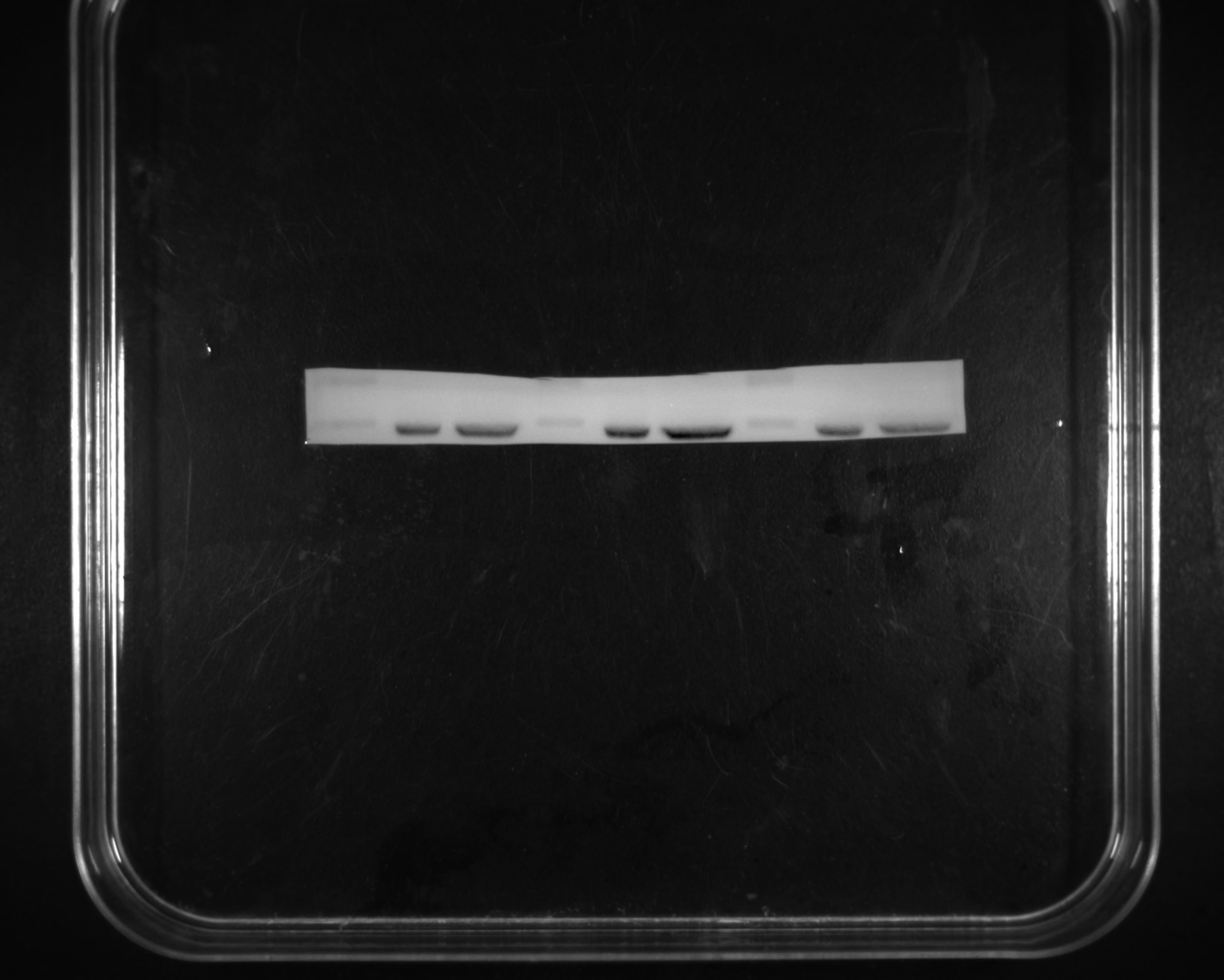

Supplement: Supplemental Information 18 — Total protein (35 µg) was extracted from the negative control HOKs (lane 1, 3, 5) and senescent HOKs (lane 2, 4, 6) (lanes were ordered from left to right). p53 (53 kDa). [file peerj-11-15158-s018.tif]

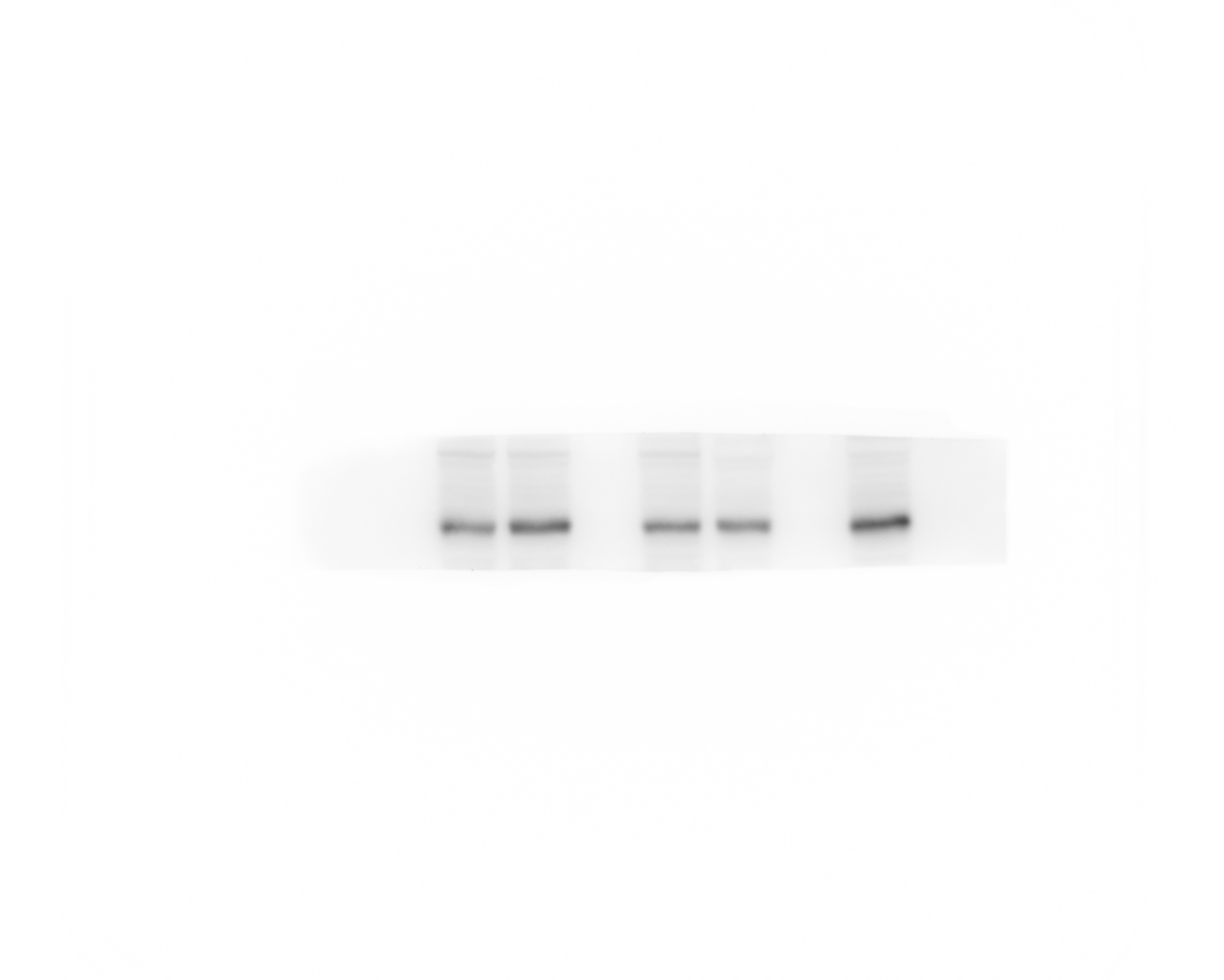

Supplement: Supplemental Information 19 — Total protein (35 µg) was extracted from the negative control HOKs (lane 1, 3, 4) and senescent HOKs (lane 2, 5) (lanes were ordered from left to right). TGF- β1 (44 kDa). [file peerj-11-15158-s019.tif]
